# ICTV Virus Taxonomy Profile: *Adenoviridae* 2022

**DOI:** 10.1099/jgv.0.001721

**Published:** 2022-03-09

**Authors:** Mária Benkő, Koki Aoki, Niklas Arnberg, Andrew J. Davison, Marcela Echavarría, Michael Hess, Morris S. Jones, Győző L. Kaján, Adriana E. Kajon, Suresh K. Mittal, Iva I. Podgorski, Carmen San Martín, Göran Wadell, Hidemi Watanabe, Balázs Harrach, ICTV Report Consortium

**Affiliations:** ^1^​ Veterinary Medical Research Institute, Budapest, Hungary; ^2^​ Hokkaido University, Sapporo, Japan; ^3^​ Umeå University, Umeå, Sweden; ^4^​ MRC-University of Glasgow Centre for Virus Research, Glasgow, UK; ^5^​ CEMIC University Hospital, CONICET, Buenos Aires, Argentina; ^6^​ University of Veterinary Medicine, Vienna, Austria; ^7^​ Naval Medical Center, San Diego, CA, USA; ^8^​ Lovelace Respiratory Research Institute, Albuquerque, NM, USA; ^9^​ Purdue University, West Lafayette, IN, USA; ^10^​ Ruđer Bošković Institute, Zagreb, Croatia; ^11^​ Centro Nacional de Biotecnología, Madrid, Spain

**Keywords:** *Adenoviridae*, *Atadenovirus*, *Aviadenovirus*, *Ichtadenovirus*, *Mastadenovirus*, *Siadenovirus*, *Testadenovirus*, ICTV Report, Taxonomy

## Abstract

The family *Adenoviridae* includes non-enveloped viruses with linear dsDNA genomes of 25–48 kb and medium-sized icosahedral capsids. Adenoviruses have been discovered in vertebrates from fish to humans. The family is divided into six genera, each of which is more common in certain animal groups. The outcome of infection may vary from subclinical to lethal disease. This is a summary of the ICTV Report on the family *Adenoviridae*, which is available at ictv.global/report/adenoviridae.

## Virion

**Fig. 1.**
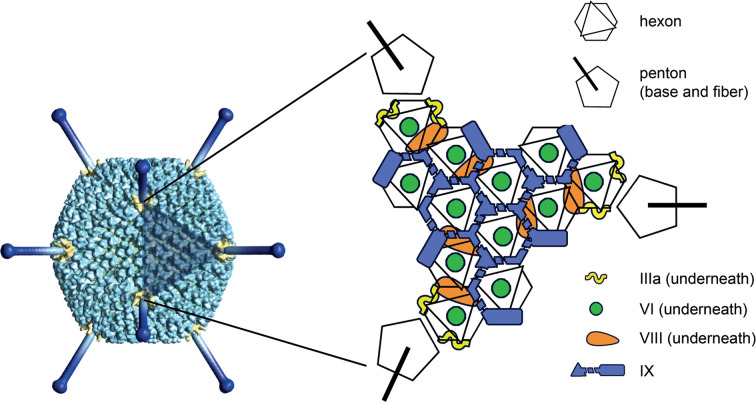
Adenovirus virion structure. Left: a model, built from a low-resolution cryo-electron microscopy reconstruction of human adenovirus 5 [9]. Yellow - penton bases, dark blue - fiber protein trimers, modelled from the crystal structure of the distal knob and the filamentous shaft [10], shaded triangle - one facet. Right: schematic of a triangular facet [1].

Adenovirus virions are non-enveloped, pseudo *T*=25 icosahedral particles. The capsid consists of 240 non-vertex (hexon) and 12 vertex capsomers (penton). The latter consist of the penton base and a protruding fiber protein trimer (Table 1, Fig. 1) [1, 2]. The minor, cementing proteins show genus-specific variation but LH3 (atadenoviruses) and protein IX (mastadenoviruses) share a capsid-binding motif [3].

**Table 1.** Characteristics of members of the family *Adenoviridae*

Example:	human adenovirus 5 (AC_000008), species *Human mastadenovirus C*, genus *Mastadenovirus*
Virion	Non-enveloped icosahedral capsid 90 nm in diameter
Genome	Linear, dsDNA of 25–48 kb with inverted terminal repeats
Replication	Nuclear
Translation	From capped, polyadenylated and often spliced transcripts
Host range	Mammals, birds, reptiles, amphibians and fish; host range varies among virus genera
Taxonomy	Realm *Varidnaviria*, kingdom *Bamfordvirae*, phylum *Preplasmiviricota*, class *Tectiliviricetes*, order *Rowavirales*; 6 genera containing >85 species

## Genome

**Fig. 2.**
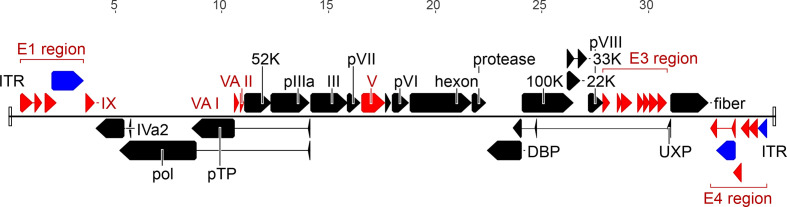
Genome organization of the mastadenovirus human adenovirus 5. Coloured arrows depict genes conserved in all genera (black), present in more than one genus (blue) or restricted to mastadenoviruses (red). Rectangles mark the inverted terminal repeats.

The genome is a single linear molecule of dsDNA of 24 630–48 395 bp [4, 5] with inverted terminal repeats of 26–721 bp (Fig. 2). A virus-encoded terminal protein is covalently linked to the 5′-end of each DNA strand.

## Replication

Entry of virus into cells occurs by attachment of the fiber trimer knob to cellular receptors followed by internalization involving interaction between the penton base and cellular α_v_ integrins [6]. After uncoating, the virus core is delivered to the nucleus, the site of virus RNA transcription, DNA replication and assembly. Infection results in the arrest of synthesis of host DNA, mRNA and proteins. Transcription by host RNA polymerase II involves both DNA strands of the virus genome. Primary transcripts are capped and polyadenylated. Complex splicing patterns govern the production of mRNA families. In primate adenoviruses, virus-associated RNA genes transcribed by cellular RNA polymerase III facilitate translation of late virus mRNAs and block the cellular interferon response.

## Pathogenesis

Human infections are usually subclinical but can, especially in immunosuppressed patients, induce acute respiratory symptoms, adenoidal–pharyngeal conjunctivitis, epidemic keratoconjunctivitis, hepatitis, acute gastroenteritis (infantile virus-caused diarrhoea), persistent interstitial infection in the kidney and haemorrhagic cystitis. Mastadenovirus infections in animals are common, but disease usually appears only when predisposing factors are present [7]. Canine adenovirus 1 is the causative agent of infectious canine hepatitis (a life-threatening disease of puppies). Skunk adenovirus 1 also infects African pigmy hedgehog, porcupine, racoon and a New World monkey. Both viruses share ancestry with bat adenoviruses [8]. In chickens, hepatitis-hydropericardium syndrome is associated with fowl adenovirus 4 and gizzard erosion with fowl adenovirus 1. Additional aviadenoviruses cause inclusion body hepatitis. The atadenovirus duck adenovirus 1 is the causative agent of egg drop syndrome in chickens, and deer adenovirus 1 infection has resulted in the death of thousands of deer in California (USA). A siadenovirus causes turkey haemorrhagic enteritis.

## Taxonomy

Current taxonomy: ictv.global/taxonomy. Genus and species demarcation is based mainly on phylogenetic criteria but also on genome organization and biological characteristics. Genus *Mastadenovirus*: >50 species (members infecting mammals); *Aviadenovirus*: >14 species (birds); *Atadenovirus*: >9 species (reptiles, birds, ruminants and marsupials); *Siadenovirus*: >7 species (birds, frogs and tortoises); *Ichtadenovirus*: 1 species (white sturgeon); *Testadenovirus*: 1 species (red-eared slider) [4, 5].

## Resources

Full ICTV Report on the family *Adenoviridae*: ictv.global/report/adenoviridae.

Sequenced adenoviruses: sites.google.com/site/adenoseq.
